# Supplementary material for: Mycobacterial dynamin-like protein IniA mediates membrane fission
Source: Nat Commun. 2019 Aug 29;10:3906. doi: 10.1038/s41467-019-11860-z (PMC6715688; doi:10.1038/s41467-019-11860-z)
Supplement: Supplementary file 1 — Supplementary Information [file 41467_2019_11860_MOESM1_ESM.pdf]

Mycobacterial dynamin-like protein IniA mediates membrane fission

M. Wang, X. Guo et al.

*Correspondence and requests for materials should be addressed to J.H. (email: huj@ibp.ac.cn) or to J.L. (email: lijun@sibcb.ac.cn)

Supplementary Figures

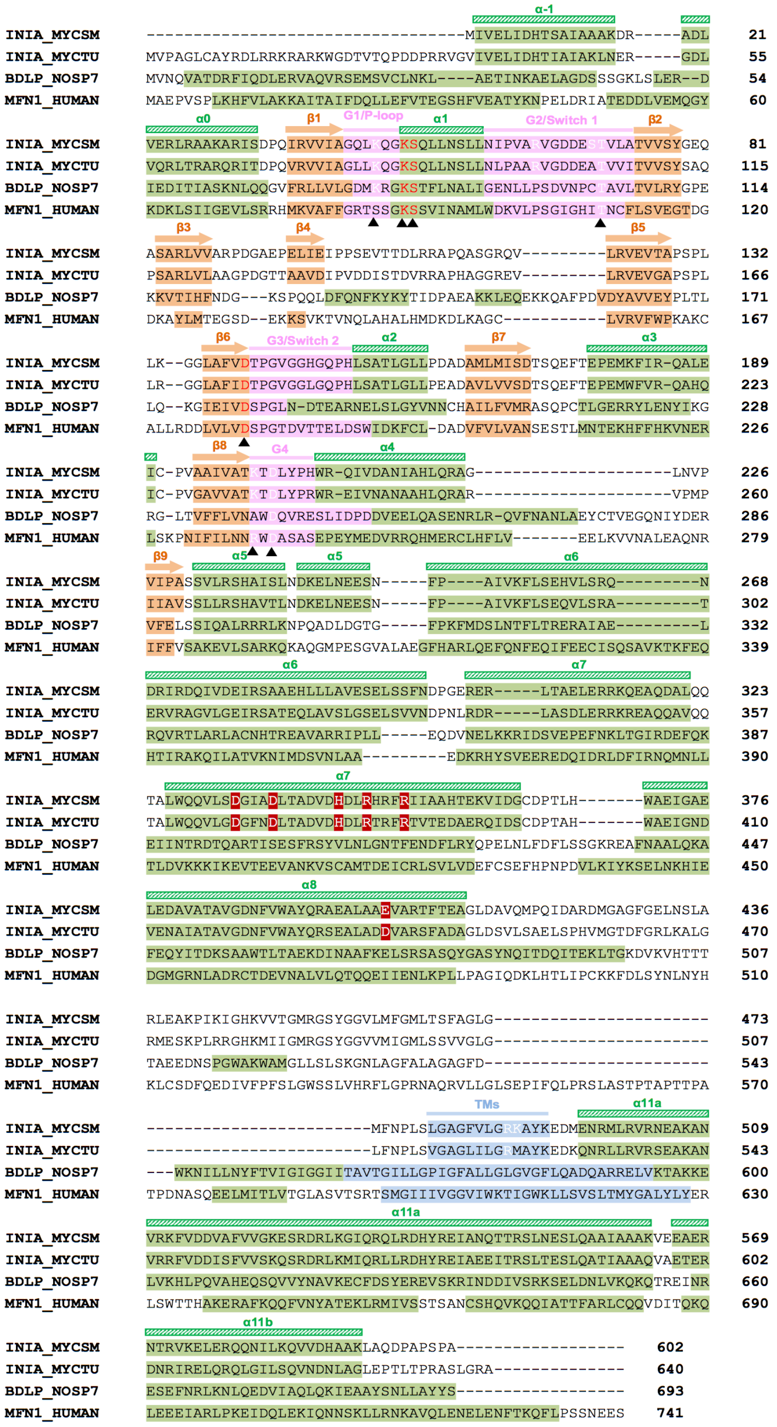

Supplementary Figure 1. Sequence alignment of IniA from *M. smegmatis*, IniA from *M. tuberculosis*, BDLP from *Nostoc punctiforme*, and MFN1 from *Homo sapiens*. Secondary structure elements and key motifs are highlighted (α helices, green; β strands, orange; GTPase motifs, pink; TMs/LI loop, light blue). Above the sequences, the α helices, β strands, and key motifs are shown by bars, arrows, and lines, respectively. Residues mentioned in this study are also highlighted or marked with black triangles.

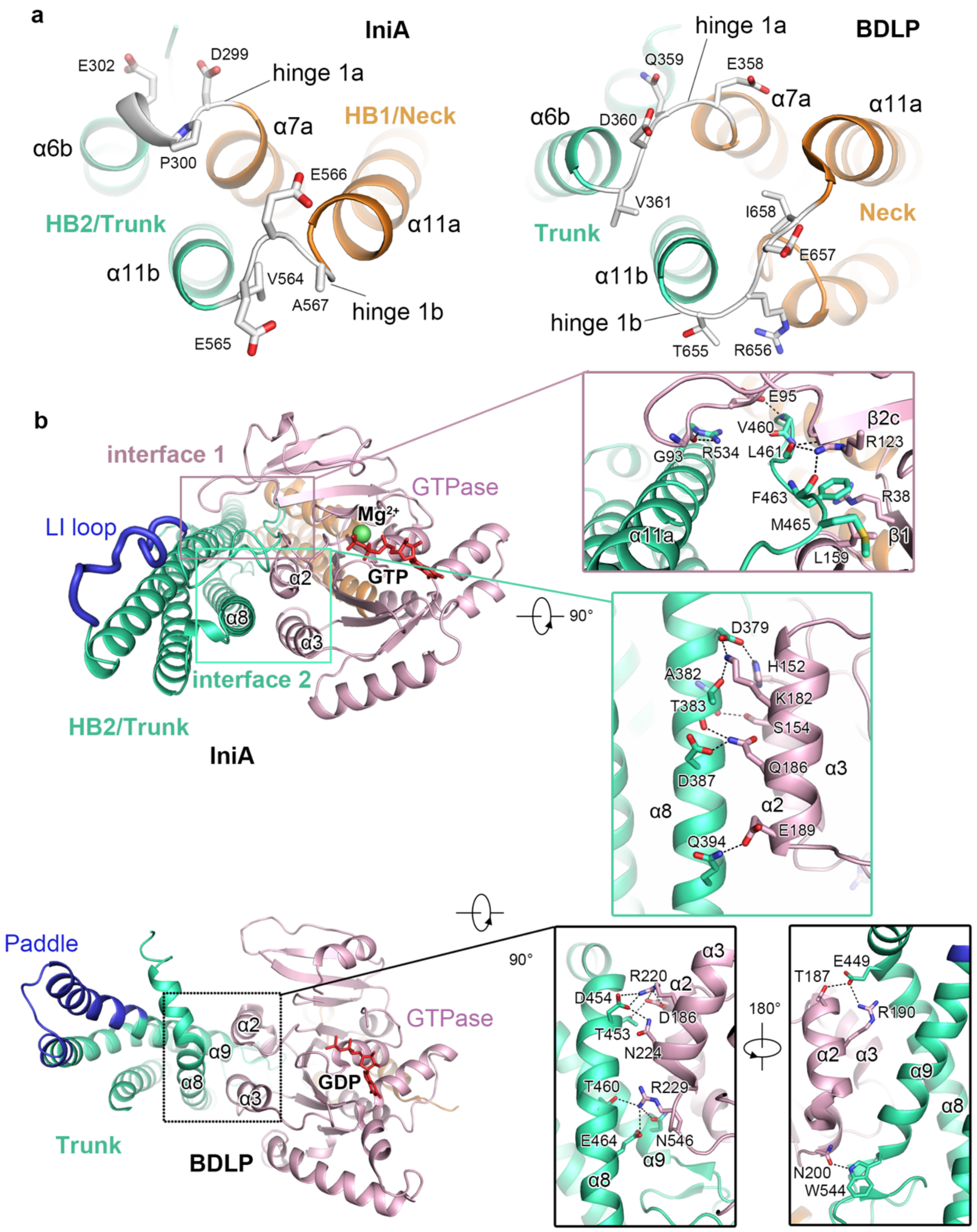

Supplementary Figure 2. Structural comparison of IniA and cyanobacteria BDLP. **a.** Comparison of the hinge regions (in white color) linking HB1/Neck and HB2/Trunk. Residues at the hinge regions are shown as sticks. **b.** Comparison of the GTPase-HB2/Trunk interfaces, marked with boxes in the left panels. The inlet panels on the right show the zoom-in view of interfaces. Polar interactions are marked with dashed lines.

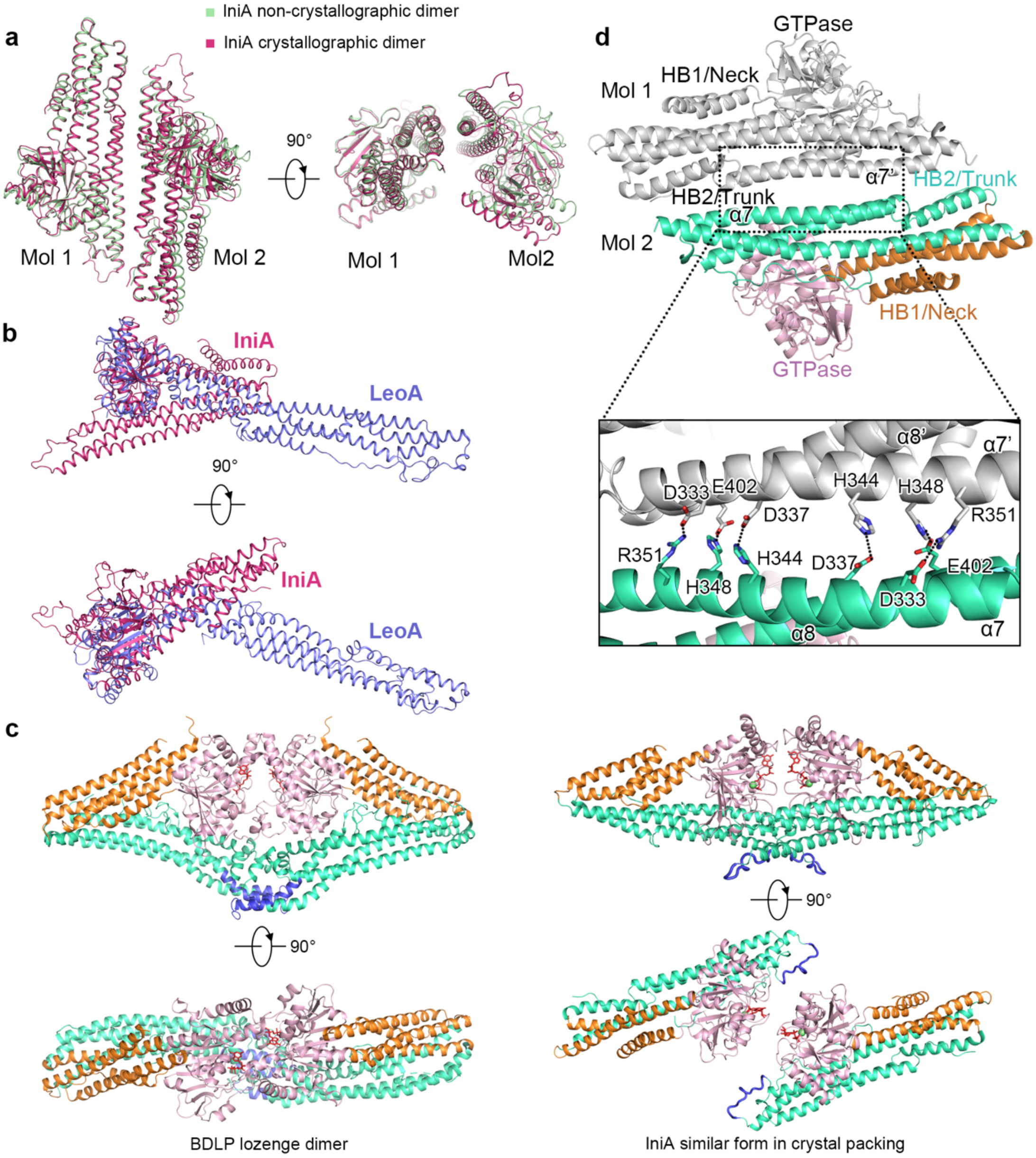

Supplementary Figure 3. Comparison of dimer forms. a. Superposition of the IniA non-crystallographic dimer in the *apo* form and crystallographic dimer in the GTP-bound form through HB2-stacking. Mol 1, monomer 1; Mol 2, monomer 2. **b.** Superposition of IniA and LeoA (PDB: 4AUR) aligned with their GTPase domains. **c.** Comparison of the BDLP lozenge dimer and similar IniA form in crystal packing. **d.** The HB2 interface in IniA crystal structures. Monomer 2 (Mol 2) is colored as in **Fig. 1b**, whereas the Mol 1 is in grey. The boxed region in the interface is zoomed in in the inset. Interactions are marked with dashed lines between corresponding residues shown as sticks.

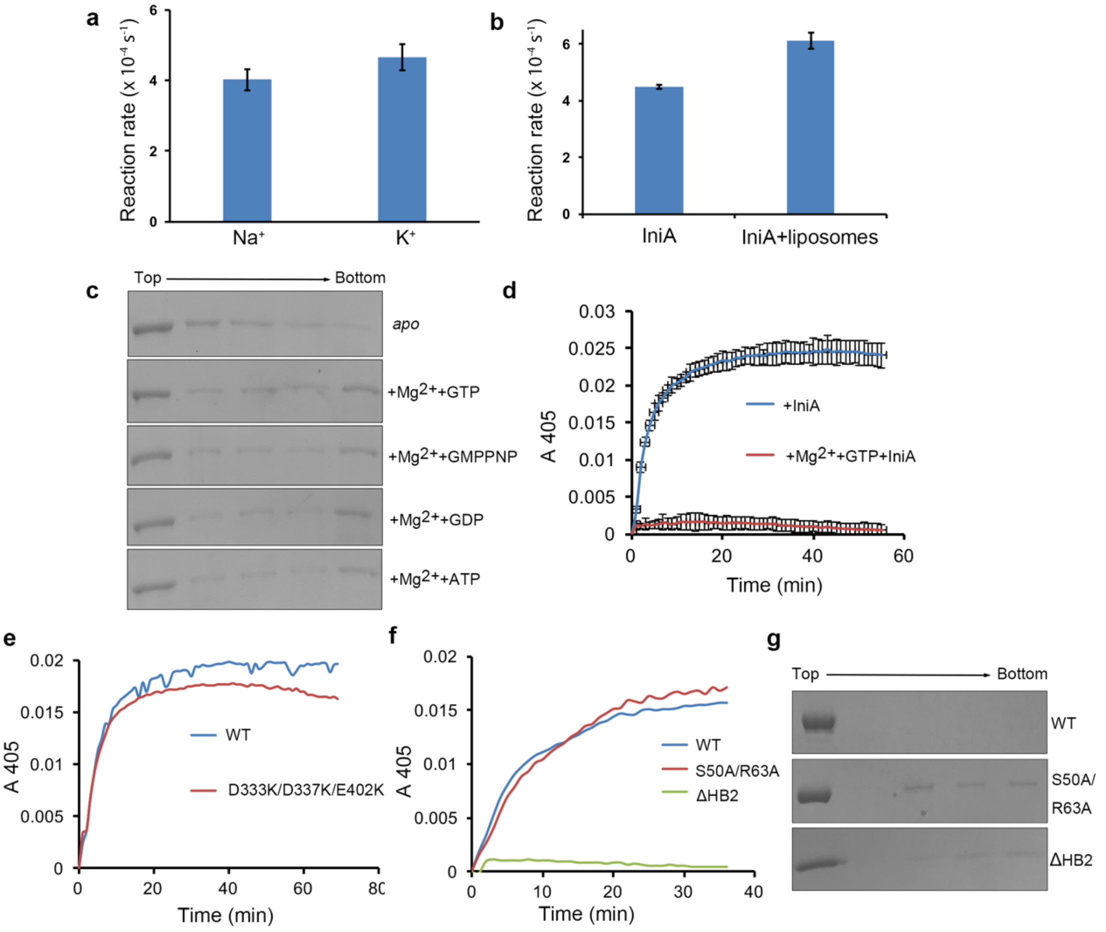

Supplementary Figure 4. Membrane association investigations of IniA. a. GTPase activity of 10 μM IniA and 0.5 mM GTP in the presence of 150 mM Na^+ or K^+. **b.** GTPase activity of 10 μM IniA and 0.5 mM GTP in the absence or presence of liposomes. Each bar is the mean and SD of three measurements. **c.** Liposome flotation assay showing the effect of 2 mM Mg^{2+} and 5 mM GTP/GMPPNP/GDP/ATP on IniA membrane association. **d.** As shown in **Fig. 4b**. Each bar is the mean and SD of three measurements. **e.** As shown in **Fig. 4b**. but test the mutant D333K/D337K/E402K. The data are representative of at least three repetitions. **f.** Liposomes tethering assay showing the nucleotide-independent oligomerization activity of wild-type IniA, S50A/R63A mutant and ΔHB2 truncation proteins. **g.** Liposome flotation assay showing the membrane binding activity of wild-type IniA, S50A/R63A mutant and ΔHB2 truncation proteins. The source data of Supplementary Fig. 4a-g are provided in the Source Data file.

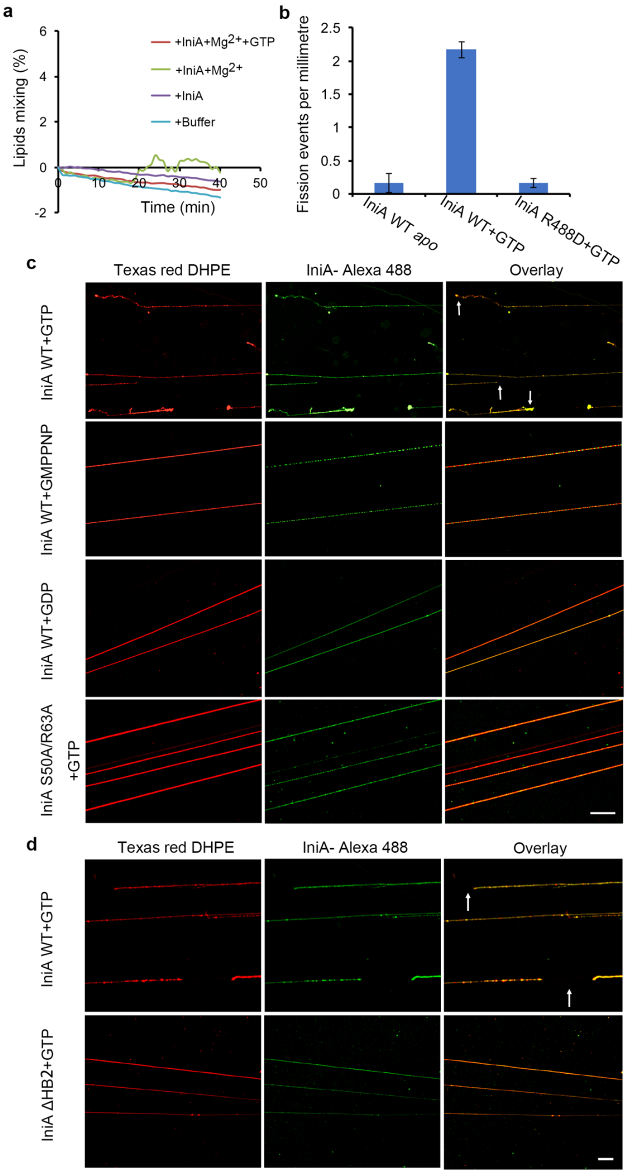

Supplementary Figure 5. Lipids mixing results and the nucleotide or mutant effect on membrane fission by IniA. a. Lipids mixing assays of 2 μM IniA in the absence or presence of 5 mM GTP and 2 mM $MgCl_2$. The data are representative of at least three repetitions. **b.** The diagram shows the statistical results in **Fig. 6a**. Each bar is the mean and SD of three measurements. **c.** As in **Fig. 6a**, SMrTs (red) were treated with wild-type IniA in the presence of 5 mM GTP/GMPPNP/GDP, and the S50A/R63A double mutant in the presence of 5 mM GTP. Protein localization and tube cleavage were monitored using confocal microscopy. The arrows indicate the cleavage sites. Scale bar, 20 μm. **d.** SMrTs (red) were treated with wild-type IniA or ΔHB2 mutant in the presence of 5 mM GTP. Protein localization and tube cleavage were monitored using confocal microscopy. The arrows indicate the cleavage sites. Scale bar, 20 μm. The source data of Supplementary Fig. 5a-b are provided in the Source Data file.

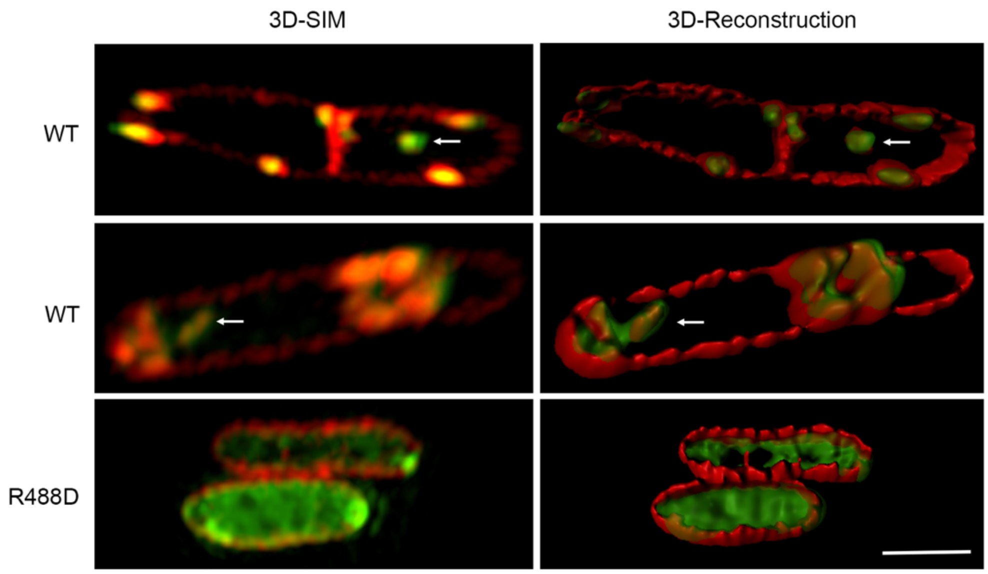

Supplementary Figure 6. IniA wt-GFP and the mutant IniA R488D-GFP fusion protein was expressed in *M. smegmatis* and their localization determined by GFP (green) and compared to that of FM4-64 (red) inserted in the membrane by 3D-SIM (left) and 3D reconstruction of z stack of images (right). Examples of intracellular vesicles and cell membrane invagination are indicated by arrows. Scale bar, 1 µm.

Supplementary Table 1. List of primers.

**Primer Name**	**Vector**	**Sequence (5'->3')**
WT-F	pET-28a	TAAGAAGGAGATATACCATGGGCGTGATCGTCGAGCTCATCGAC
WT-R	pET-28a	ATCCGGCACCGAGTCCGGCCAAGCTTGCGGCCGCACTCGA
WT-F1	PMV261	TACTTCCAATCCAATGCTGTGATCGTCGAGCTCATCGAC
WT-R1	PMV261	TTATCCCACCCAAATGGGCCGGACTCGGTGC
WT-F2	S9-vector	GATGTACTCAAGGAGGTGATCGTCGAGCTCAT
WT-R2	S9-vector	ATGGTGGTGATGGTGTCAGGCCGGACTCGGTGCCG

**Mutant Name**	**Sequence (5'->3')**
Ybbr-F	GAATTTATTGCTAGTAAGCTTGCGGCACTCGAGCACCACC
Ybbr-R	AGCTTACTAGCAATAAATTCAAGAGAATCCTTGGCCGGACTCGGTGC
D333/337K-2F	GCCGACGTCGACCACGACCTGCGGCACCGGTTC
D333/337K-2R	GGTCGACGTCGGCGGTCAGCTTGGCGATACCCTTGCTGAG
E402K-F	AACGCGCCGAGGCGCTGGCGGCCAAGGTGGCGCGGA
E402K-R	AACTTCGTATGGGCCTATCAACGCGCCGAGGCGCTGGCG
K46A-F	GCCGGGCAGCTCGCGCAGGGCAAGAG
K46A-R	GATGACGACGCGGATCTGCGGAT
K49A-F	CTCAAGCAGGGCGCGAGCCAGCTGCT
K49A-R	CTGCCCGGCGATGACGACGCGGA
S50A-F	AAGCAGGGCAAGGCCCAGCTGCTCAA
S50A-R	GAGCTGCCCGGCGATGACGACGC
R63A-F	ACTCGCTGCTCAACATCCCGGTGGCGGCCGTCGGTGACGA
R63A-R	CGGGATGTTGAGCAGCGAGTTGAGCAGCTGGCTCTTGCCC
V485S-F	GGCGCGGGTTTCTCGCTGGGGCGCAAGGCC
L486S-F	GGCGCGGGTTTCGTGTCGGGGCGCAAGGCC
V485/ L486S-2R	GAGCGACAACGGGTTGAACATGCCCAGACC
R488D-F	TCGTGCTGGGGGACAAGGCCTACAAGGAGG
K489E-F	TCGTGCTGGGGCGCGAGGCCTACAAGGAGG
R488D/K489E-2R	AACCCGCGCCGAGCGACAACGGGTTGAACA
K492E-F	GCGCAAGGCCTACGAGGAGGACATGGAGAA
K492E-R	CCCAGCACGAAACCCGCGCCGAGCGACAAC
R498D-F	AAGGCCTACAAGGAGGACATGGAGAACGACATGCTG
R498D-R	CCTCCTTGTAGGCCTTGCGCCCCAGCACGA
Δ480-492-F	GGAGGCGGAGGTTCAGGTGGCGGAGGTAGTGAGGACATGGAGAACCGCATGCTG
Δ480-492-R	ACTACCTCCGCCACCTGAACCTCCGCCTCCCGACAACGGGTTGAACATGC
ΔHB2-F	TCGTGCTGGGGCGCAAGGCCTACAAGGTCGAGGAGGCCGAACGCAATAC
ΔHB2-R	GAATCGGAACTGTCATCGTTCAACGACCTCGGCGCGGGTTTCGTGCTGGGGCGCAAGGCC

Supplementary Table 2. List of Bacterial Sources.

**Bacterial and Virus Strains**	**SOURCE**	**IDENTIFIER**
E. coli BL21 (DE3)	ATCC	ATCCPTA-5073
M. smegmatis mc^2155	ATCC	ATCC:700084
