# Supplementary material for: Mycobacterial dynamin-like protein IniA mediates membrane fission
Source: Nat Commun. 2019 Aug 29;10:3906. doi: 10.1038/s41467-019-11860-z (PMC6715688; doi:10.1038/s41467-019-11860-z)
Supplement: Supplementary file 3 — Reporting Summary [file 41467_2019_11860_MOESM3_ESM.pdf]

nature research

Corresponding author(s): Junjie Hu, Jun Li

☐ Initial submission ☐ Revised version ☒ Final submission

Life Sciences Reporting Summary

Nature Research wishes to improve the reproducibility of the work that we publish. This form is intended for publication with all accepted life science papers and provides structure for consistency and transparency in reporting. Every life science submission will use this form; some list items might not apply to an individual manuscript, but all fields must be completed for clarity.

For further information on the points included in this form, see Reporting Life Sciences Research. For further information on Nature Research policies, including our data availability policy, see Authors & Referees and the Editorial Policy Checklist.

Experimental design

1. Sample size

Describe how sample size was determined.	*Describe the statistical methods that were used to predetermine sample size OR if no sample-size calculation was performed, describe how sample sizes were chosen and provide a rationale for why these sample sizes are sufficient.*

2. Data exclusions

Describe any data exclusions.	No data were excluded from data analyses.

3. Replication

Describe whether the experimental findings were reliably reproduced.	All attempts at replication were successful.

4. Randomization

Describe how samples/organisms/participants were allocated into experimental groups.	*Describe how samples were allocated to groups. If allocation was not random, describe how covariates were controlled. If this is not relevant to your study, explain why.*

5. Blinding

Describe whether the investigators were blinded to group allocation during data collection and/or analysis.	*Describe the extent of blinding used during data acquisition and analysis. If blinding was not possible, describe why OR explain why blinding was not relevant to your study.*

Note: all studies involving animals and/or human research participants must disclose whether blinding and randomization were used.

6. Statistical parameters

For all figures and tables that use statistical methods, confirm that the following items are present in relevant figure legends (or in the Methods section if additional space is needed).

n/a	Confirmed	
☐	☐	The exact sample size (*n*) for each experimental group/condition, given as a discrete number and unit of measurement (animals, litters, cultures, etc.)
☐	☐	A description of how samples were collected, noting whether measurements were taken from distinct samples or whether the same sample was measured repeatedly
☐	☒	A statement indicating how many times each experiment was replicated
☐	☐	The statistical test(s) used and whether they are one- or two-sided (note: only common tests should be described solely by name; more complex techniques should be described in the Methods section)
☐	☐	A description of any assumptions or corrections, such as an adjustment for multiple comparisons
☐	☐	The test results (e.g. *P* values) given as exact values whenever possible and with confidence intervals noted
☐	☒	A clear description of statistics including central tendency (e.g. median, mean) and variation (e.g. standard deviation, interquartile range)
☐	☒	Clearly defined error bars

See the web collection on statistics for biologists for further resources and guidance.

Software

Policy information about availability of computer code

7. Software

Describe the software used to analyze the data in this study.	Standard software for structural determination, including PHENIX, COOT and PyMOL, was described and cited in methods.

For manuscripts utilizing custom algorithms or software that are central to the paper but not yet described in the published literature, software must be made available to editors and reviewers upon request. We strongly encourage code deposition in a community repository (e.g. GitHub). *Nature Methods* guidance for providing algorithms and software for publication provides further information on this topic.

Materials and reagents

Policy information about availability of materials

8. Materials availability

Indicate whether there are restrictions on availability of unique materials or if these materials are only available for distribution by a for-profit company.	No unique materials were used.

9. Antibodies

Describe the antibodies used and how they were validated for use in the system under study (i.e. assay and species).	*For all antibodies, as applicable, provide supplier name, catalog number, clone name, and lot number. Also describe the validation of each primary antibody for the species and application, noting any validation statements on the manufacturer's website, relevant citations, antibody profiles in online databases, or data provided in the manuscript OR state that no antibodies were used.*

10. Eukaryotic cell lines

a. State the source of each eukaryotic cell line used.	*Provide information on cell line source(s) OR state that no eukaryotic cell lines were used.*
b. Describe the method of cell line authentication used.	*Describe the authentication procedures for each cell line used OR declare that none of the cell lines used have been authenticated OR state that no eukaryotic cell lines were used.*
c. Report whether the cell lines were tested for mycoplasma contamination.	*Confirm that all cell lines tested negative for mycoplasma contamination OR describe the results of the testing for mycoplasma contamination OR declare that the cell lines were not tested for mycoplasma contamination OR state that no eukaryotic cell lines were used.*
d. If any of the cell lines used are listed in the database of commonly misidentified cell lines maintained by ICLAC, provide a scientific rationale for their use.	*Provide a rationale for the use of commonly misidentified cell lines OR state that no commonly misidentified cell lines were used.*

Animals and human research participants

Policy information about studies involving animals; when reporting animal research, follow the ARRIVE guidelines

11. Description of research animals

Provide details on animals and/or animal-derived materials used in the study.	*For laboratory animals, report species, strain, sex and age OR for animals observed in or captured from the field, report species, sex and age where possible OR state that no animals were used.*

Policy information about studies involving human research participants

12. Description of human research participants

Describe the covariate-relevant population characteristics of the human research participants.	*Provide all relevant information on human research participants, such as age, gender, genotypic information, past and current diagnosis and treatment categories, etc. OR state that the study did not involve human research participants.*
