# Supplementary material for: Mycobacterial dynamin-like protein IniA mediates membrane fission
Source: Nat Commun. 2019 Aug 29;10:3906. doi: 10.1038/s41467-019-11860-z (PMC6715688; doi:10.1038/s41467-019-11860-z)
Supplement: Supplementary file 5 — Description of Additional Supplementary Files [file 41467_2019_11860_MOESM5_ESM.docx]

**Title: Supplementary Movie 1** **Membrane fission by IniA.**
**Description:** SMrTs (red) were treated with wildtype IniA (labeled green) in the presence of 5 mM GTP. Protein localization and tube cleavage were monitored using confocal microscopy.
